# Diphtheria from an Unclean Cellar

**Published:** 1888-09

**Authors:** Clarissa Potter


### DIPHTHERIA FROM AN UNCLEAN CELLAR.

"I could not understand why that entire family of seven children should be stricken with putrid diphtheria, till I had occasion to go into their cellar;" a friend said, who had been summoned to help care for the sick and dying children of a neighbor.

"We always thought the Wrights, with their trim, white-washed fences and out-buildings, their neatly kept door-yard and garden, the evident constant warfare, against filth and slatternliness in any form, the most intelligent and cleanly of families in our community, and I wondered what possible breeding place for malignant diphtheria could lurk about that home, till I went into the cellar.

When I opened the stairway door, a horrible stench of decaying vegetables and tainted brine rushed up from the unventilated, loathsome pit below, that they cellar. The air was so heavy with mold and stagnant impurities that the flame of the candle I carried, flickered and lapped over, as though a weight had been laid on it.

Hardly had I stepped from the bottom stair, before my feet struck a slippery, slimy chute of rotten pumpkin, and I went down into the dreadful mush that sent out its pestilential whiffs from the very depths of its putrification.

The candle still burned, and after hastily rising from this unexpected tobogganing arcoss the cellar bottom, I held the sickly flame high and low, scanning well that breeding nest of diphtheria and other fearful germs, before cutting the slices of salted pork, for which I had been sent to bind upon the poor little, swollen, choked throats, up stairs.

Walls, green with mold and fungi; decayed and decaying vegetables everywhere; a slosh of rotted apples oozing their pungent juices from the bloated staves of a dozen barrels; a great bin of frozen, then thawed, potatoes, that to stir meant development of gas, powerful enough to run an electric plant if odor is power. Under the stairs a heap of pumpkins had been stored in the late autumn, that decaying bloat—months before—had hoisted and rolled apart, some of the mushing, sliding spheres falling directly in the pathway and making the slippery chute that had unbalanced and mired me; and in every corner, putrifying stacks of turnips and cabbages sending out their penetrating, loathsome breaths.

The cellar was as dark as a coal pit, the little three-pane light under the dining-room windows being buried under the winter banking that late May still found unmoved.

The vile gases and stagnant air, thick with dreadful odors and digease germs, had no outlet, of escape from the cellar, only by stealthily filtering through every possible cranny and seam of the heavy timbered coiling into the living and sleeping rooms overhead, and by strong rushes up the stairway, whenever the the opening cellar door stirred a current upward."

And, still, those parents wondered why their seven young children, whom they thought to cherish and protect from every harm, should be stricken with diphtheria, and called it one of the most mosterious of God's providences when they were called to lay two of their darlings under the sod.—CLARISSA POTTER in *The Housewife.*

